# Mitochondrial DNA Analysis Clarifies Taxonomic Status of the Northernmost Snow Sheep (*Ovis nivicola*) Population

**DOI:** 10.3390/life11030252

**Published:** 2021-03-18

**Authors:** Arsen V. Dotsev, Elisabeth Kunz, Veronika R. Kharzinova, Innokentiy M. Okhlopkov, Feng-Hua Lv, Meng-Hua Li, Andrey N. Rodionov, Alexey V. Shakhin, Taras P. Sipko, Dmitry G. Medvedev, Elena A. Gladyr, Vugar A. Bagirov, Gottfried Brem, Ivica Medugorac, Natalia A. Zinovieva

**Affiliations:** 1 L.K. Ernst Federal Science Center for Animal Husbandry, 142132 Moscow, Russia; veronika0784@mail.ru (V.R.K.); rodiand@yandex.ru (A.N.R.); alexshahin@mail.ru (A.V.S.); elenagladyr@mail.ru (E.A.G.); vugarbagirov@mail.ru (V.A.B.); gottfried.brem@agrobiogen.de (G.B.); 2 Population Genomics Group, Department of Veterinary Sciences, LMU Munich, 80539 Munich, Germany; Elisabeth.Kunz@gen.vetmed.uni-muenchen.de (E.K.); Ivica.Medjugorac@gen.vetmed.uni-muenchen.de (I.M.); 3 Institute for Biological Problems of Cryolithozone, 677000 Yakutsk, Russia; imo-ibpc@yandex.ru; 4 College of Animal Science and Technology, China Agricultural University, Beijing 100193, China; lvfenghua@cau.edu.cn (F.-H.L.); menghua.li@ioz.ac.cn (M.-H.L.); 5 A.N. Severtsov Institute of Ecology and Evolution of the Russian Academy of Sciences, 119071 Moscow, Russia; sipkotp@mail.ru; 6 Department of Game Management and Bioecology, Irkutsk State University of Agriculture, 664038 Irkutsk, Russia; dmimedvedev@yandex.ru; 7 Institut für Tierzucht und Genetik, University of Veterinary Medicine (VMU), A-1210 Vienna, Austria

**Keywords:** wild sheep, bighorn, taxonomy, mtDNA, cytochrome b, Yakut snow sheep, *Ovis nivicola lydekkeri*

## Abstract

Currently, the intraspecific taxonomy of snow sheep (*Ovis nivicola*) is controversial and needs to be specified using DNA molecular genetic markers. In our previous work using whole-genome single nucleotide polymorphism (SNP) analysis, we found that the population inhabiting Kharaulakh Ridge was genetically different from the other populations of Yakut subspecies to which it was usually referred. Here, our study was aimed at the clarification of taxonomic status of Kharaulakh snow sheep using mitochondrial cytochrome b gene. A total of 87 specimens from five different geographic locations of Yakut snow sheep as well as 20 specimens of other recognized subspecies were included in this study. We identified 19 haplotypes, two of which belonged to the population from Kharaulakh Ridge. Median-joining network and Bayesian tree analyses revealed that Kharaulakh population clustered separately from all the other Yakut snow sheep. The divergence time between Kharaulakh population and Yakut snow sheep was estimated as 0.48 ± 0.19 MYA. Thus, the study of the mtDNA *cytb* sequences confirmed the results of genome-wide SNP analysis. Taking into account the high degree of divergence of Kharaulakh snow sheep from other groups, identified by both nuclear and mitochondrial DNA markers, we propose to classify the Kharaulakh population as a separate subspecies.

## 1. Introduction

Snow sheep *(Ovis nivicola)* (Video S1) is an endemic species in northeastern Siberia and the Russian Far East. Along with the North American mountain sheep (bighorn) *(Ovis canadensis)* and Dall sheep *(Ovis dalli)*, it is referred to the subgenus *Pachyceros* of the genus *Ovis* [1]. The intraspecific taxonomy of snow sheep is highly controversial and currently remains insufficiently studied [2,3,4]. While some scientists believe that snow sheep should not be divided into subspecies, but rather be considered as a subspecies of North American wild sheep [5], others distinguish up to seven subspecies [6]. According to the Chernyavsky classification [4], which is also officially recognized by the International Council for Game and Wildlife Conservation (CIC), four subspecies are distinguished including Kamchatka *(O. n. nivicola)*, Koryak *(O. n. koriakorum)*, Putorana *(O. n. borealis)* and Yakut *(O. n. lydekkeri)* [7].

To date, the taxonomic divisions of snow sheep *(O. nivicola)* into subspecies have been based on the morphological differences between individuals. However, morphological characteristics are influenced by both the origin of the animals and the environmental conditions (food supply, temperature, etc.). Therefore, the use of this approach can be misleading in the determination of population structure. Thus, differences between individuals of different origins can be leveled while they are in similar environmental conditions. On the contrary, animals of the same or similar genetic origin can develop distinctive characteristics due to their adaptation to various environmental conditions.

The investigation of DNA polymorphisms makes it possible to identify “true” genetic differences between individuals, and it is becoming more widely used in research on issues of biological systematics [8]. The most commonly used methodological approach is to study the polymorphisms of mitochondrial DNA (mtDNA). The advantage of mtDNA is the absence of recombination and the maternal type of inheritance as well as the possibility to extract mtDNA from the small amounts of biological samples since animal cells contain numerous copies of it. For example, using mtDNA, three species of tahr were assigned to separate monotypic genera-*Hemitragus jemlahicus*, *Nilgiritragus hylocrius* and *Arabitragus jayakari*, while by morphological characteristics they were classified as the different subspecies of the same species [9]. The development of DNA chips for simultaneous analysis of several thousand or even hundreds of thousands of single-nucleotide polymorphisms (SNPs) in the genome of farm animals made it possible to use this highly informative tools to study the genomes of related wild species [10,11], including the bighorn [12] and snow sheep [13]. In the latter work, it was revealed that populations of Yakut snow sheep *(O. n lydekkeri)*, traditionally considered as a single subspecies, were represented by individuals with different origins. In particular, a different origin of the snow sheep inhabiting the most northern area of the snow sheep habitats—the Kharaulakh Ridge—from other populations of the Yakut snow sheep was established [13]. The additional research using other types of DNA variability is necessary to confirm the status of Kharaulakh sheep as an independent subspecies. Because of broad applications in phylogenetic studies and recognitions of results by the scientific community, the mtDNA polymorphisms are the most suitable type of sequence variability for this purpose. The use of mitochondrial DNA is of particular interest in the study of *O. nivicola* due to its maternal heritability. It is a well-known fact that snow sheep females are much less migratory than the males. As a rule, ewes remain in the habitat where they were born while rams can migrate for distances up to 100–150 km to search for females [3].

Thus, our present work was aimed at clarifying the taxonomic status of the Kharaulakh population of snow sheep based on the analysis of the polymorphism of the mitochondrial cytochrome B (*cytb*) gene.

## 2. Materials and Methods

**Figure 1:**
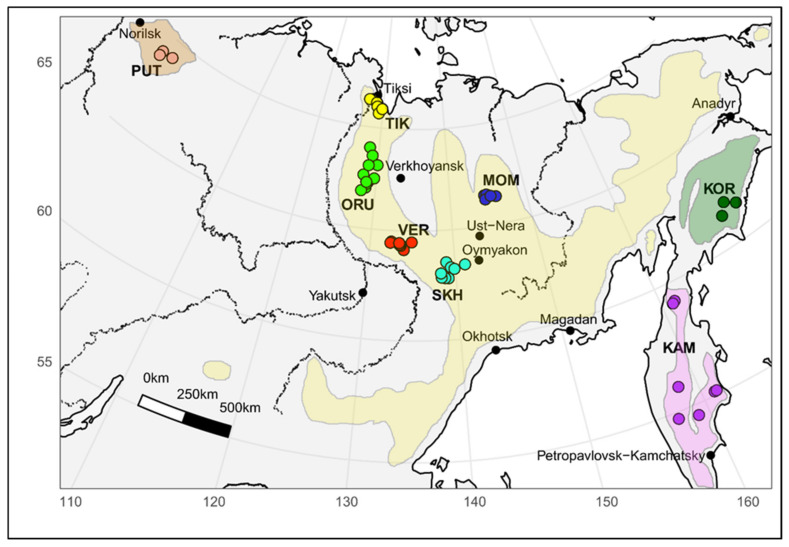
Map with sampling sites of snow sheep used in this study and the area of the species habitat. TIK = Kharaulakh Ridge–Tiksi Bay, ORU = Orulgan, VER = Central Verkhoyansk, SKH = Suntar-Khayata, MOM = Momsky, KAM = Kamchatka snow sheep, KOR = Koryak, PUT = Putorana.

The animals of five different geographic locations of Yakut snow sheep *(O. n. lydekkeri)* including Kharaulakh Ridge-Tiksi bay (TIK, *n* = 21), Orulgan (ORU, *n* = 25), Central Verkhoyansk (VER, *n* = 23), Suntar-Khayata (SKH, *n* = 11) and Momsky (MOM, *n* = 7) ridges as well as the samples of the other three most recognized subspecies, including Kamchatka (KAM, *n* = 9), Koryak (KOR, *n* = 8), Putorana (PUT, *n* = 3), were selected for the study (Figure 1). Muscle tissue samples of snow sheep were collected under permits issued by the Department of Hunting of the Republic Sakha (Yakutia) during scientific expeditions. Some samples were taken from trophy hunters and indigenous peoples’ representatives, who are licensed to hunt snow sheep for personal consumption according to the Federal Law of the Russian Federation. We were able to obtain only a few samples, which are suitable for deriving DNA of the appropriate quality, from the Red Book Putorana subspecies from animals which died due to natural causes.

DNA extraction was carried out using Nexttec columns (Nexttec Biotechnology GmbH, Leverkusen, Germany) in accordance with the manufacturer’s recommendations. The whole sequences of *cytb* gene of Yakut snow sheep were determined by Sanger sequencing of two overlapping fragments (overlap area of about 50 bp). The *cytb* sequences of Kamchatka, Koryak, and Putorana snow sheep were defined using next generation sequencing (NGS) technology [14]. For this purpose, three overlapping mtDNA fragments were amplified (overlapping region more than 290 bp) with a length of 6.5, 5.7, and 6.7 kb. The obtained polymerase chain reaction (PCR) products were purified and used to prepare the libraries for sequencing, which were then sequenced by 100 bp paired-end procedure on a HiSeq 1500 (Illumina). To verify that the results obtained from Sanger sequencing and NGS technology are comparable, a part of Yakut snow sheep samples (*n* = 20) were sequenced by the two methods. It was shown that all the compared sequences were identical. Mitos WebServer [15] was used to annotate the mitochondrial genome. The *cytb* sequence was recovered from the complete mtDNA sequence after its alignment, performed using the MUSCLE algorithm [16] in the MEGA 7.0.26 software [17].

The *cytb* gene sequences of North American wild sheep were downloaded from the GenBank NCBI database (www.ncbi.nlm.nih.gov (accessed on 15 December 2020)): Dall sheep *(O. dalli)* (ODA, *n* = 3), Rocky Mountain bighorn *(O. canadensis canadensis)* (OCC, *n* = 8) and desert bighorn sheep *(O. canadensis nelsoni)* (OCN, *n* = 11) were added to the final dataset. A complete list of these samples with their GenBank NCBI accession numbers is presented in Table 1.

To construct a median joining network [20], PopART 1.7 software [21] was used. For Bayesian phylogenetic reconstruction, one sample with the most frequent haplotype from each population was selected. The population from Orulgan Ridge was represented by two samples belonging to the central Verkhoyansk-VER/ORU and to the Kharaulakh Range-TIK/ORU haplotypes. The construction of the Bayesian phylogenetic tree was carried out using the program BEAST 2.5 [22] with subsequent visualization in FigTree 1.4.2 (http://tree.bio.ed.ac.uk/software/figtree (accessed on 15 December 2020)). Determination of the best models of evolution was carried out separately for each nucleotide in the program PartitionFinder 2 [23] using the Akaike information corrected criterion (AICc) [24]. The most optimal were the evolutionary models HKY + I, HKY and HKY + G, respectively, for the first, second and third codons of the *cytb* gene. Calculations of pairwise *F*_ST_ as well as AMOVA analysis were carried out in the program Arlequin 3.5.2.2 [25]. Genetic distances based on the Kimura-2-parameter model [26] were calculated using the MEGA 7.0.26 software. Further, based on these distances, a Neighbor-Net phylogenetic tree was constructed in the SplitsTree 4.14.6 program [27]. DnaSP 6.12.01 program [28] was used to calculate genetic diversity parameters: number of polymorphic sites (S), average number of nucleotide differences (K), number of haplotypes (H), haplotype diversity (Hd), nucleotide diversity (π). We also tested the hypothesis of population expansion calculating Fu’s neutrality statistic Fs [29] and Tajima’s D [30] test in DnaSP 6.12.01. Demographic histories of snow sheep populations were inferred by pairwise mismatch distribution analyses [31] and computed under a constant population size model in DnaSP 6.12.01.

Divergence time was estimated using BEAST 2.5 software. To calibrate molecular clock, we added a prior to the model assuming that the two species of the outgroup–*O. canadensis* and *O. dalli* have been split 1.5 million years ago (MYA). This calibration node was retrieved as median time of divergence from the TimeTree web resource (http://www.timetree.org (accessed on 15 December 2020)) [32].

R packages “maps” [33] and “ggplot2” [34] were used to create maps with sampling sites.

## 3. Results and Discussion

In order to clarify the taxonomic status of the Kharaulakh population of snow sheep, which, according to the results of SNP genome-wide analysis was proposed as an independent subspecies [13], we performed a study of the whole cytochrome B (*cytb*) gene sequence in four mostly recognized subspecies of snow sheep including the Yakut (*O. n. lydekkeri*), Kamchatka (*O. n. nivicola*), Koryak (*O. n. koriakorum*) and Putorana (*O. n. borealis*). The *cytb* sequences of Dall sheep (*O. dalli*) and two subspecies of the bighorn sheep (*O. canadensis canadensis*) and (*O. canadensis nelsoni*) were included in the final dataset as outgroups.

In total, we identified 19 haplotypes in 107 samples of snow sheep. Moreover, *O. n. koriakorum* and *O. n. borealis*, as well as the *O. n. lydekkeri* population from the Momsky Ridge were each represented by a single haplotype. The values of genetic diversity parameters (Table 2) in Yakut snow sheep populations were consistent with data obtained in a study based on SNP markers. Thus, haplotype diversity (Hd) on the Verkhoyansk Range increased southwards, from 0.381 ± 0.101 in Kharaulakh to 0.746 ± 0.098 in the Suntar-Khayata population. The lowest values of nucleotide diversity (π) and the average number of nucleotide differences (K) were also observed in the Kharaulakh population of snow sheep.

The results of Tajima’s D and Fu’s Fs tests were statistically insignificant in all the populations, so we could not reject the null hypothesis, which indicates deviations from neutrality and suggests recent population expansion.

**Figure 2:**
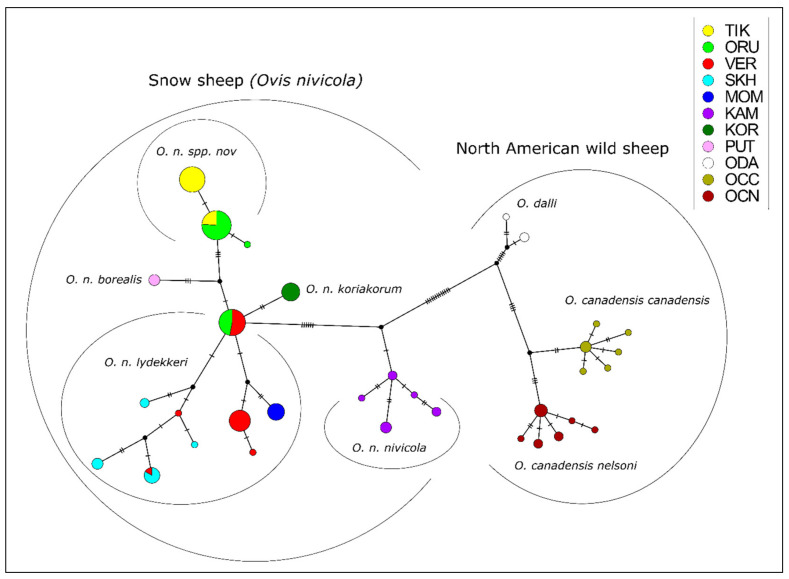
Median joining network of snow sheep (*Ovis nivicola*) and North American wild sheep haplotypes, based on analysis of mtDNA *cytb* gene polymorphism. The diameter of the circle corresponds to the number of individuals belonging to this haplotype. The number of transverse lines indicates the number of nucleotide substitutions. The black circles at the branching points of the network are hypothetical haplotypes. TIK = Kharaulakh Ridge–Tiksi Bay, ORU = Orulgan, VER = Central Verkhoyansk, SKH = Suntar-Khayata, MOM = Momsky, KAM = Kamchatka, KOR = Koryak, PUT = Putorana, ODA = Dall sheep, OCC = Rocky Mountain bighorn, OCN = desert bighorn sheep.

The median joining network (Figure 2) showed that the group inhabiting the mountains of Kamchatka–*O. n. nivicola* was the most separated from the others and was the closest to the historical ancestor of the modern snow sheep. All the other samples were derived from a single haplotype currently occurring in the central Verkhoyansk Range—VER and on the Orulgan Ridge—ORU. Among the samples from the Kharaulakh Ridge, we identified two haplotypes, which differed from each other by a single mutation. One of these haplotypes was also found in animals from the Orulgan Ridge. Thus, the presence in ORU population of haplotypes which are differed from each other by four nucleotide substitutions confirms the admixed origin of this population, as it was revealed by the study on multiple SNP markers [13]. A hypothetical haplotype, linking the population from the Kharaulakh Ridge with the Putorana snow sheep, was established.

Genetic distances based on the Kimura-2-parameter model (Table 3) confirmed the most genetic distance of the Kamchatka snow sheep from the other studied groups.

**Figure 3:**
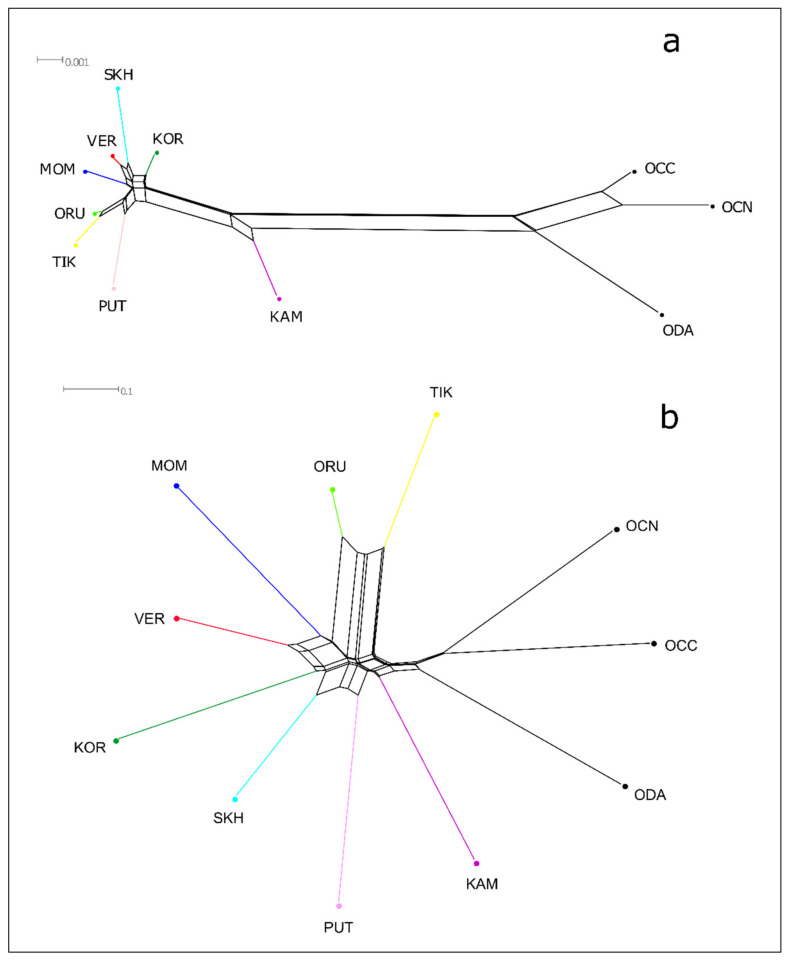
Neighbor-Net tree of snow sheep (*Ovis nivicola*) and North American wild sheep, based on genetic distances based on the Kimura-2-parameter model (**a**) and pairwise *F*_ST_ (**b**). TIK = Kharaulakh Ridge–Tiksi Bay, ORU = Orulgan, VER = Central Verkhoyansk, SKH = Suntar-Khayata, MOM = Momsky, KAM = Kamchatka, KOR = Koryak, PUT = Putorana, ODA = Dall sheep, OCC = Rocky Mountain bighorn, OCN = desert bighorn sheep.

The Central Verkhoyansk, the Suntar-Khayata, and the Momsky populations had similar genetic distances with the Kharaulakh population (0.005, 0.008, 0.005, respectively) and with the population of Putorana Plateau (0.005, 0.007, 0.006), and lower with Koryak (0.003, 0.005, 0.004). Pairwise *F*_ST_ values revealed high genetic differentiation between the populations of snow sheep. The lowest *F*_ST_ values were observed between TIK and ORU–0.438 and the highest between TIK and KOR-0.959. It was shown that *F*_ST_ values between TIK and populations of *O. n. lydekkeri* (VER, SKH, MOM) ranged from 0.831 (TIK and VER) to 0.950 (TIK and MOM) and were significantly higher than within the other Yakut snow sheep populations—from 0.560 (VER and SKH) to 0.741 (SKH and MOM). These results are consistent with the study of Yakut snow sheep based on whole-genome SNP markers [13]. Based on genetic distances and pairwise *F*_ST_, we constructed Neighbor-Net trees (Figure 3).

**Figure 4:**
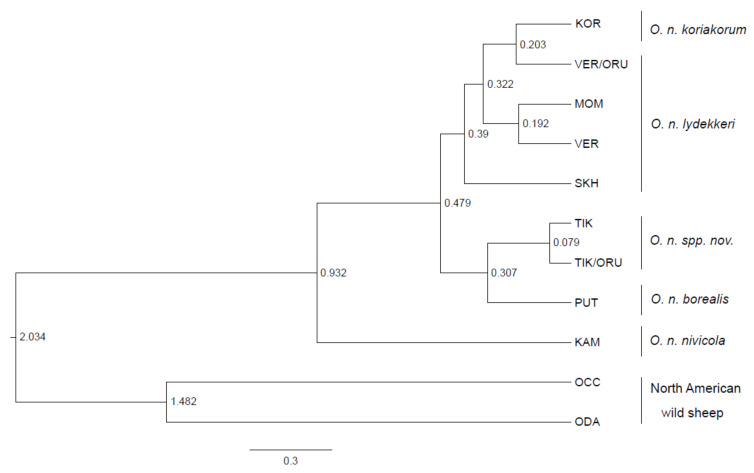
Bayesian phylogenetic tree of snow sheep (*Ovis nivicola*) and North American wild sheep indicating divergence time (in MYA) estimates based on the mtDNA *cytb* gene.

Similar conclusions can be drawn from the analysis of the Bayesian phylogenetic tree (Figure 4): two Kharaulakh haplotypes formed a clade with the Putorana snow sheep.

All the populations of Yakut (VER, SKH, MOM) along with Koryak snow sheep were placed in another clade. The Kamchatka population was the most distant from all the other snow sheep groups. To estimate approximate divergence time between the populations of snow sheep, we calibrated molecular clock, considering pairwise divergence time for *O. canadensis* and *O. dalli*–1.5 million years ago (MYA), calculated as a median from the previous studies [1,18,35,36] and given on the web resource TimeTree [32]. According to our model, we obtained the divergence time between *O. nivicola* and North American wild sheep as being around 2 MYA, which agreed with the median time for these species indicated on the web resource TimeTree–1.94 MYA. The Kharaulakh population (TIK) diverged from the closest group from Putorana Plateau (PUT) around 0.3 ± 0.13 MYA and from Yakut snow sheep populations (VER, SKH, MOM)–0.48 ± 0.19 MYA. The most distant Kamchatka population was split from all the other populations of snow sheep around 0.93 ± 0.35 MYA. All the above mentioned clades were supported with high posterior probability values: TIK-PUT–0.91, TIK-Yakut snow sheep–1, KAM-all the other populations of snow sheep–1 (Figure S1).

The results of AMOVA, which was conducted for four populations of *O. n. lydekkeri*: TIK, VER, SKH and MOM, further supported genetic differentiation between the populations of Yakut snow sheep with significant variation of 78.8% (Table 4). Only 21.2% of genetic variation was found within populations. ORU was not included in this test due to its admixed origin.

**Figure 5:**
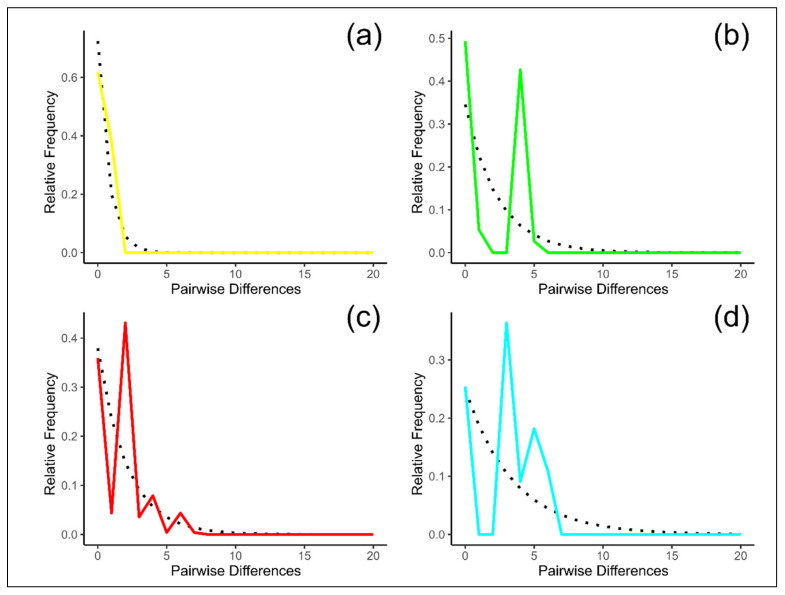
Mismatch distribution for the populations of *Ovis nivicola*. The dash line represents the expected distribution under the constant population size model and the solid line is the observed pairwise difference. (**a**) TIK, (**b**) ORU, (**c**) VER and (**d**) SKH population. TIK = Kharaulakh Ridge–Tiksi, ORU = Orulgan, VER = Central Verkhoyansk, SKH = Suntar-Khayata ridges.

The mismatch distribution (the distribution of the number of pairwise differences between sequences) revealed that in TIK the observed curve agreed with the expected constant population size model (Figure 5). For all the other populations, mismatch distributions were multimodal. These results may reflect the fact that the TIK population, which inhabits the northernmost periphery of the species area, evolved independently, without admixture with other groups. All the other populations could survive during periods of glaciation in refugia and subsequently expand their areas, mixing with other populations. For example, the population from Orulgan Ridge (ORU) was formed by admixture of the Kharauakh population with groups from the Central Verkhoyansk Range, as it was shown by both nuclear [13] and mitochondrial DNA studies.

According to our research results, we can divide populations of snow sheep into three major groups. The first, and the most distant group, is presented by sheep from Kamchatka. The second group joints the Putorana and Kharaulakh populations, and the third one includes Yakut snow sheep along with representatives of the Koryak subspecies. Our results are not entirely consistent with the traditional subdivision of snow sheep, which was based mainly on the morphological characteristics. Thus, the Kharaulakh population should not be considered as *O. n. lydekkeri* and, rather, be classified as a separate subspecies. The status of Koryak subspecies should be further explored using more samples and different types of genetic markers.

Finally, the results of our study should lead to reassessment of snow sheep protection programs in Yakutia. At present, the most numerous Yakut snow sheep subspecies is protected only in the Momsky Natural Park and resource reserves: “Orulgan-Sis” (Orulgan Ridge), “Verkhneindigirsky” (Chersky Ridge), “Kele” (Central part of Verkhoyansk Range). In these territories, environments for the preservation of natural resources (by limiting economic activity) and conditions necessary for the protection of plants and animals are created [37]. The studied population of snow sheep on the Kharaulakh Ridge is protected only on the territory of the federal state natural reserve “Ust-Lensky”, where hunting for this species is limited to certain periods and lasts from 1 August to 30 November. Indigenous peoples are allowed to hunt snow sheep for traditional activities also only during these periods. The recognition of the Kharaulakh population as a separate subspecies will make it possible to estimate the census size and organize new conservation areas in breeding and feeding grounds. The establishment of a new resource reserve in the Kharaulakh Ridge is essential for conservation management of the northernmost snow sheep population.

## 4. Conclusions

Our study of the whole *cytB* sequence in the four most recognized subspecies of Asian snow sheep (*Ovis nivicola*) showed the most genetic distance of the Kamchatka population (*O. n. nivicola*). The haplotypes of all other populations were originated from a single haplotype currently found in the central Verkhoyansk Range and the Orulgan Ridge. It was shown that, in terms of the number of nucleotide substitutions, the Kharaulakh population differs from this “main” Yakut haplotype even more than the officially recognized Putorana and Koryak subspecies. The Orulgan population has the admixture origin and is represented by two major haplotypes, differing from each other by four nucleotide substitutions. Thus, the study of the mtDNA *cytb* sequences confirmed the results of genome-wide SNP research. Taking into account the high degree of divergence of Kharaulakh snow sheep from other groups, identified by both nuclear and mitochondrial DNA markers, we propose to classify the Kharaulakh population as a separate subspecies. The results of our study can be used in biodiversity conservation programs.

## Figures and Tables

**Table 1:** List of North American wild sheep mtDNA *cytb* gene sequences downloaded from the GenBank NCBI database (www.ncbi.nlm.nih.gov (accessed on 15 December 2020)).

#	Species	*n*	GenBank Accession Number	References
1	Rocky Mountain bighorn sheep (*Ovis canadensis canadensis*)	8	EU365985, EU366063, EU366064, EU366065, EU366066, EU366067, FJ936176, FJ936177	Rezaei H.R. et al. [18]
2	Desert bighorn sheep (*Ovis canadensis nelsoni*)	11	EU366059, EU366060, EU366061, EU366062, FJ936178, FJ936179, FJ936180, FJ936181, FJ936182, FJ936183,	Rezaei H.R. et al. [18]
			HM222706	Naidu A. et al. [19]
3	Dall sheep (*Ovis dalli*)	3	MH779627	Dotsev A. et al. [14]
			EU365992, FJ936184	Rezaei H.R. et al. [18]

**Table 2:** Genetic diversity and neutrality indices of snow sheep populations calculated from nucleotide sequence of mitochondrial *cytb* gene.

Population	*n*	S	K (±SD)	H	Hd (±SD)	π (±SD)	Tajima’s D	Fu’s Fs
TIK	21	1	0.381 ± 0.375	2	0.381 ± 0.101	0.00033± 0.00037	0.65593	0.94374
ORU	25	5	1.893 ± 1.117	3	0.507 ± 0.075	0.00166 ± 0.00109	1.23135	3.39988
VER	23	7	1.636 ± 1.002	5	0.640 ± 0.065	0.00144 ± 0.00098	−0.43378	0.34957
SKH	11	8	3.018 ± 1.703	4	0.746 ± 0.098	0.00265 ± 0.00169	0.43451	1.79212
MOM	7	0	0	1	0	0	-	-
KAM	9	7	2.722 ± 1.592	5	0.861 ± 0.087	0.00239 ± 0.00158	0.25402	−0.16693
KOR	8	0	0	1	0	0	-	-
PUT	3	0	0	1	0	0	-	-

Population: TIK = Kharaulakh Ridge—Tiksi Bay, ORU = Orulgan, VER = Central Verkhoyansk, SKH = Suntar-Khayata, MOM = Momsky, KAM = Kamchatka, KOR = Koryak, PUT = Putorana; *n*—number of samples; S—number of polymorphic sites; K—average number of nucleotide differences; SD—standard deviation; H—number of haplotypes; Hd—haplotype diversity; π—nucleotide diversity; Tajima’s D—value of Tajima’s neutrality test, Fu’s Fs—the value of Fu’s neutrality test.

**Table 3:** Genetic distances between the studied snow sheep populations and North American wild sheep.

Population	TIK	ORU	VER	SKH	MOM	KOR	KAM	PUT	OCC	OCN	ODA
TIK	*	0.002	0.005	0.008	0.005	0.006	0.012	0.006	0.025	0.022	0.025
ORU	**0.438**	*	0.004	0.006	0.004	0.004	0.010	0.005	0.024	0.021	0.024
VER	**0.831**	**0.570**	*	0.004	0.003	0.003	0.009	0.005	0.023	0.021	0.024
SKH	**0.850**	**0.661**	**0.560**	*	0.006	0.005	0.011	0.007	0.025	0.025	0.028
MOM	**0.950**	**0.689**	**0.651**	**0.741**	*	0.004	0.010	0.006	0.024	0.022	0.025
KOR	**0.959**	**0.718**	**0.665**	**0.714**	**1.000**	*	0.009	0.005	0.023	0.021	0.024
KAM	**0.919**	**0.813**	**0.804**	**0.771**	**0.868**	**0.865**	*	0.011	0.020	0.020	0.021
PUT	**0.950**	**0.704**	**0.741**	**0.708**	**1.000**	**1.000**	**0.834**	*	0.026	0.025	0.028
OCC	**0.972**	**0.924**	**0.933**	**0.913**	**0.967**	**0.968**	**0.904**	**0.958**	*	0.012	0.013
OCN	**0.972**	**0.934**	**0.941**	**0.926**	**0.966**	**0.967**	**0.912**	**0.959**	**0.769**	*	0.006
ODA	**0.981**	**0.929**	**0.935**	**0.899**	**0.981**	**0.982**	**0.884**	**0.966**	**0.876**	**0.886**	*

Below diagonal are shown pairwise *F*_ST_ and above diagonal genetic distances (based on the Kimura-2-parameter model). The significant *F*_ST_ values are represented in bold (*p* < 0.05). Population: TIK = Kharaulakh Ridge–Tiksi Bay, ORU = Orulgan, VER = Central Verkhoyansk, SKH = Suntar-Khayata, MOM = Momsky, KAM = Kamchatka, KOR = Koryak, PUT = Putorana, ODA = Dall sheep, OCC = Rocky Mountain bighorn, OCN = desert bighorn sheep. Asterisk: zero.

**Table 4:** The results of AMOVA for the Yakut populations of *O. nivicola* based on the cytochrome b gene.

Source of Variation	d.f.	SS	VC	V%
Among populations	3	105.035	2.36458	78.8
Within populations	58	36.9	0.63621	21.2
Total	61	141.935	3.00080	

d.f = degrees of freedom; SS = sum of squares; VC = variance components; V% = percent of variation.

## Data Availability

The *cytb* gene sequences of snow sheep obtained for this study were deposited in GenBank NCBI database (www.ncbi.nlm.nih.gov (accessed on 15 December 2020)). Accession numbers: MW736905 - MW737011.

## Supplementary Materials

The following are available online at https://www.mdpi.com/2075-1729/11/3/252/s1, Figure S1: Bayesian phylogenetic tree of snow sheep (*Ovis nivicola*) and North American wild sheep indicating posterior probability values, Video S1: A representative of the northernmost population of snow sheep (Kharaulakh Ridge). Video: Bendersky E.V.
